# Exploring the Role of Cerebrospinal Fluid and Serum Mid-Regional Pro-Adrenomedullin in Tick-Borne Encephalitis: A Pilot Study

**DOI:** 10.3390/diagnostics16010095

**Published:** 2025-12-27

**Authors:** Gabriela Trojan, Anna Moniuszko-Malinowska, Joanna Oklińska, Wioletta Pawlak-Zalewska, Ewelina Kruszewska, Agnieszka Kulczyńska-Przybik, Barbara Mroczko, Piotr Czupryna

**Affiliations:** 1 Department of Infectious Diseases and Neuroinfections, Medical University of Bialystok, 15-089 Bialystok, Poland; annamoniuszko@op.pl (A.M.-M.); joanna.oklinska@gmail.com (J.O.); viola103@tlen.pl (W.P.-Z.); kruszewska.ewelina@gmail.com (E.K.); avalon-5@wp.pl (P.C.); 2 Department of Neurodegeneration Diagnostics, Medical University of Bialystok, 15-089 Bialystok, Poland; agnieszka.kulczynska-przybik@umb.edu.pl; 3 Department of Biochemical Diagnostics, Medical University of Bialystok, 15-089 Bialystok, Poland; barbara.mroczko@umb.edu.pl

**Keywords:** adrenomedullin, MR-proADM, tick-borne encephalitis, cerebrospinal fluid, biomarker, neuroinflammation, neuroinfection

## Abstract

**Background**: Adrenomedullin (ADM) is a multifunctional peptide with vasoregulatory, antimicrobial, and anti-inflammatory properties. Its stable fragment, mid-regional pro-adrenomedullin (MR-proADM), is a validated biomarker in sepsis and systemic infections, but its role in viral neuroinfections remains unexplored. Tick-borne encephalitis (TBE), caused by the tick-borne encephalitis virus (TBEV), is a major viral infection of the central nervous system (CNS) associated with long-term neurological sequelae. This study aimed to assess MR-proADM levels in cerebrospinal fluid (CSF) and serum of patients with TBE and to evaluate their diagnostic utility and pathophysiological significance. **Methods**: This retrospective observational study included 20 patients with confirmed TBE and 14 non-infectious neurological controls. MR-proADM concentrations were measured in paired CSF and serum samples using an ELISA assay. Statistical analyses included group comparisons (Mann–Whitney U test), correlation analyses (Spearman’s r), and receiver operating characteristic (ROC) curve evaluation. **Results**: Serum MR-proADM levels at baseline (SER1) were significantly lower in TBE patients compared with controls (*p* = 0.0197). The CSF/serum MR-proADM ratio differed significantly between groups (*p* = 0.0063) and showed the best diagnostic performance (AUC = 0.816, 95% CI 0.63–0.93; sensitivity 79%, specificity 80%). MR-proADM concentrations in CSF correlated with total CSF protein (r = 0.53), suggesting an association with blood–CSF barrier dysfunction. Strong reproducibility was observed for serum MR-proADM between sampling points (r = 0.83). **Conclusions**: MR-proADM levels in CSF and serum are altered in patients with TBE, indicating its potential as a biomarker of CNS infection and inflammation. The CSF/serum MR-proADM ratio may serve as a sensitive indicator of blood–CSF barrier involvement, while decreased serum levels may reflect impaired systemic neuroprotective response. These findings highlight a possible role of ADM in neuroimmune regulation during viral encephalitis and warrant validation in larger prospective studies.

## 1. Introduction

Adrenomedullin (ADM) has attracted growing attention in biomedical research due to its diverse physiological functions and emerging clinical utility as a biomarker, particularly in conditions associated with infection and inflammation [1,2,3,4]. Its stable fragment, mid-regional pro-adrenomedullin (MR-proADM), has been validated as a reliable biomarker in infectious diseases and sepsis, offering both diagnostic and prognostic value [5]. Beyond its measurable diagnostic potential, ADM exerts complex biological effects, acting as an antimicrobial neuropeptide with notable structural and functional similarities to classical antimicrobial peptides (AMPs), including comparable size, amphiphilicity, and net cationic charge [6,7]. These properties confer a broad antimicrobial spectrum in vitro and support a protective role for ADM in maintaining tissue integrity under microbial threat [8].

Importantly, ADM exhibits dual functionality—it not only contributes to pathogen defense but also attenuates excessive inflammatory responses [8]. This balance between antimicrobial and anti-inflammatory activity is particularly relevant within the central nervous system (CNS), where limiting immune-mediated tissue injury is critical for maintaining neuronal homeostasis [6]. Elevated plasma and cerebrospinal fluid (CSF) MR-proADM levels have been consistently observed in patients with severe infections, supporting its utility as a biomarker in both systemic and CNS-specific inflammatory conditions [5]. In a multicenter study, CSF MR-proADM concentrations, as well as CSF-to-blood ratios, were significantly higher in infectious compared to non-infectious neurological diseases, indicating its potential as a discriminative diagnostic tool in acute CNS infections [5].

Beyond bacterial infections, ADM appears to play an important role in viral pathogenesis [4,9]. Evidence shows that ADM concentrations increase in a range of viral infections, suggesting a broader function in host immune regulation and tissue response [6]. In addition, interactions between ADM and cytoskeletal proteins influence neuronal microtubule dynamics, implicating ADM in pathways of neuroinflammation, apoptosis, and calcium dysregulation [10,11]. These findings point to a complex interplay between ADM, immune modulation, and neuronal function—mechanisms particularly relevant in viral neuroinfections. However, despite extensive research on MR-proADM in bacterial and systemic infections, its role in viral neuroinfections, including TBE, has not yet been characterized.

Tick-borne encephalitis (TBE) represents one of the most significant viral neuroinfections in Europe and Asia, caused by the tick-borne encephalitis virus (TBEV). Three subtypes of the TBEV are recognized: European, Siberian, and Far-Eastern, which differ in their degree of virulence [12,13]. In Poland, only the European subtype is present [14]. The disease is endemic in at least 27 countries, with 10,000–15,000 reported cases annually and evidence of geographic expansion into previously non-endemic areas [12,15,16]. In Poland TBE is endemic, with 200–300 cases reported each year. About 90% of them occur in two provinces bordering the Baltic States [13,15,17]. It should be noted that no causal (etiological) treatment exists for TBE. The only management we can offer patients is symptomatic therapy. An effective vaccine is available and prevents severe forms of the disease; however, it is not mandatory. It is only recommended in endemic regions, for individuals traveling to endemic areas, as well as for forest workers, soldiers, and farmers.

Clinically, TBE manifests as a biphasic illness: an initial febrile phase followed by a neurological phase involving meningitis, meningoencephalitis, or meningoencephalomyelitis [15,17,18]. Severe disease requiring intensive care occurs in up to 16% of hospitalized patients, with mortality ranging from 0.9% to 2.9%, depending on the viral subtype and patient characteristics [16]. Importantly, long-term sequelae are common—up to 50% of survivors experience postencephalitic syndrome, including cognitive and neuropsychiatric impairments that persist for months or years [16,19,20,21].

Given the growing incidence of TBE, the considerable burden of long-term neurological complications, and the diagnostic challenges associated with differentiating infectious from post-infectious inflammatory changes, there is a pressing need for reliable biomarkers reflecting disease activity and CNS involvement.

Considering ADM’s established role in infection, inflammation, and neuroprotection, and the diagnostic utility of MR-proADM in CNS infections, we hypothesized that MR-proADM could serve as a potential biomarker in TBE. Therefore, the present study aimed to assess MR-proADM concentrations in the CSF of patients with TBE and evaluate their relationship with disease severity and neurological outcomes, to explore its potential as a diagnostic and prognostic biomarker in viral neuroinfection.

## 2. Materials and Methods

### 2.1. Study Design and Ethical Approval

This retrospective observational study used archival biological material collected in 2023–2024. The study protocol was approved by the Local Bioethics Committee (Approval No. APK.002.583.2023). All patients had given written informed consent for the use of their samples in research.

### 2.2. Participants and Samples

Thirty-four participants were included: 20 patients with confirmed TBE and 14 non-infectious controls. Diagnosis of TBE was based on clinical presentation, CSF inflammatory parameters and serology for TBEV (presence of specific IgM/IgG in serum and/or CSF) in accordance with established case definitions. The TBE cohort was subdivided by disease severity into severe (meningoencephalitis/meningoencephalomyelitis; *n* = 9) and mild (meningitis; *n* = 11). Controls were subjects undergoing lumbar puncture for acute neurological symptoms (i.e., headache) in whom infectious, definite inflammatory, vascular and neurodegenerative diseases were excluded after clinical, laboratory and neuroimaging evaluation. None of the patients had been vaccinated against TBE. In none of these cases was a neurological diagnosis ultimately established.

A total of 105 biological samples were processed (paired CSF and serum samples, with follow-up sampling in the TBE group approximately one month after admission)—sample handling: centrifugation, aliquoting and storage at −80 °C until assay. For clarity, the following nomenclature was adopted to indicate the timing of sample collection: CSF1 and SER1 denote cerebrospinal fluid and serum collected simultaneously during the first hospital admission, corresponding to the acute phase of the disease. CSF2 and SER2 refer to cerebrospinal fluid and serum obtained from the same patients during a follow-up hospitalization approximately one month after discharge, performed to monitor disease progression.

### 2.3. MR-proADM Measurement

MR-proADM concentrations in serum and CSF were measured using the Human MR pro-ADM (Mid-Regional pro-Adrenomedullin) ELISA Kit (FineTest, Wuhan Fine Biotech Co., Wuhan, China) according to the manufacturer’s protocol. Samples and standards were incubated on microplates coated with capture antibody; detection was performed using biotin-conjugated antibody and HRP-streptavidin, with colorimetric readout (TMB) and absorbance read at 450 nm. Concentrations were calculated from a standard curve and reported in pmol/L (or the units returned by the kit). All assays were performed in duplicate; inter- and intra-assay CVs were monitored according to kit recommendations.

### 2.4. Other Laboratory Measurements

Routine CSF parameters (glucose, total protein, albumin, cell count and differential), serum biochemistry (ALT, AST, creatinine, D-dimers), and inflammatory markers (CRP) were measured in the hospital laboratory by standard methods and were available in the database.

### 2.5. Statistical Analysis

Data cleaning and analysis were performed using Statistica 13.3 (StatSoft) and Medcalc 11.3. Continuous variables are presented as medians and interquartile ranges (non-normal distributions) or means ± SD if approximately normal. Group comparisons (TBE vs. control and subgroups) were performed using the Mann–Whitney U test (two-sided) for continuous variables and Fisher’s exact test or χ^2^ for categorical variables. Correlations between continuous variables were assessed with Spearman’s rank correlation coefficient. Receiver-operating characteristic (ROC) curve analysis (DeLong method for AUC SE) was used to assess diagnostic performance of serum and CSF MR-proADM and derived ratios; optimal cut-offs were selected by inspection of sensitivity/specificity and likelihood ratios, reporting AUC, 95% CI, and *p*-value for AUC = 0.5. A two-sided *p* < 0.05 was considered statistically significant. AUC (Area Under the Curve) represents a model’s ability to distinguish between positive and negative classes across all thresholds, with AUC near 1 being excellent and 0.5 being random chance.

A cut-off point was suggested on the basis of best specificity and sensibility.

## 3. Results

Thirty-four participants (20 TBE patients and 14 controls) were included in the analysis, yielding a total of 105 processed samples (paired cerebrospinal fluid and serum specimens). Baseline demographic characteristics did not differ significantly between groups. The mean age in the control group was 38 years (median 40), compared with 43 years (median 43) in the TBE group.

In group comparisons, serum MR-proADM levels at baseline (SER1) were significantly lower in TBE patients than in controls (Mann–Whitney U test; Z = −2.33, *p* = 0.0197). The CSF-to-serum MR-proADM concentration ratio also differed significantly between groups (Z = 2.73, *p* = 0.0063). Comparison of serum MR-proADM concentrations between patients with mild and severe TBE did not reveal statistically significant differences, likely due to the limited sample size (severe *n* = 9; mild *n* = 11). Mean MR-proADM concentrations across study groups are summarized in Table 1.

Correlation analyses demonstrated a strong association between CSF MR-proADM levels measured at baseline and follow-up (CSF1 vs. CSF2; Spearman r = 0.67). In addition, CSF MR-proADM concentrations at follow-up (CSF2) showed a moderate correlation with total CSF protein levels (r = 0.53), suggesting a relationship between intrathecal MR-proADM expression and blood–CSF barrier dysfunction or inflammation. Serum MR-proADM measurements were highly reproducible between time points (SER1 vs. SER2; r = 0.83).

Receiver operating characteristic (ROC) analysis revealed fair diagnostic performance of serum MR-proADM (SER1) in differentiating TBE patients from controls (AUC = 0.759, 95% CI 0.572–0.894, *p* = 0.0089). A cut-off value of ≤475.3 pmol/L yielded a sensitivity of 85% and a specificity of 72.7%.

**Figure 1:**
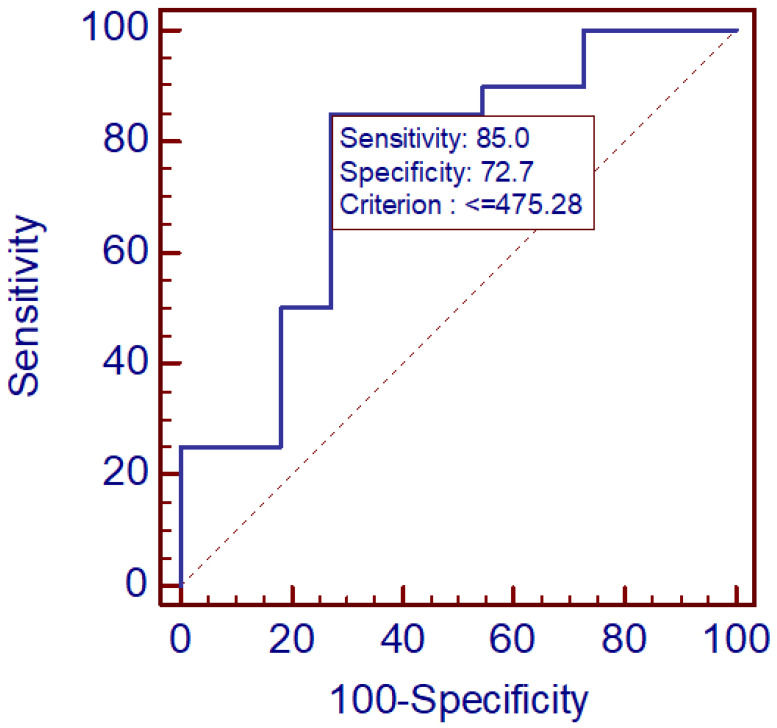
ROC analysis of serum MR-proADM.

**Figure 2:**
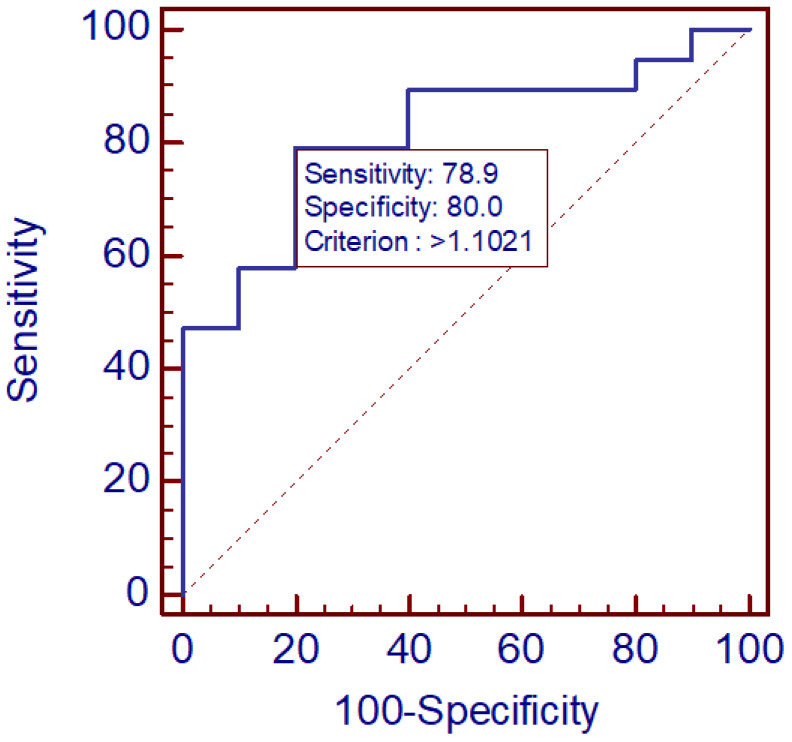
ROC curve for CSF/serum MR-proADM ratio.

For the CSF/serum MR-proADM ratio, diagnostic performance was higher (AUC = 0.816, 95% CI 0.629–0.934, *p* = 0.0001). A cut-off value >1.10 provided 79% sensitivity and 80% specificity, indicating favorable discriminative potential for clinical application. The results are illustrated in Figure 1 and Figure 2. Overall, these findings suggest that the CSF/serum MR-proADM ratio may offer greater diagnostic utility than serum measurements alone.

## 4. Discussion

This study provides the first evidence that MR-proADM concentrations in both CSF and serum are altered in patients with TBE, supporting its potential role as a biomarker reflecting the extent of CNS involvement. We found significantly lower serum MR-proADM levels (SER1) in TBE compared with non-infectious controls, as well as a significant difference in the CSF/serum ratio. ROC analysis demonstrated moderate to good diagnostic accuracy for differentiating TBE from other neurological disorders, with an AUC of 0.76 for serum MR-proADM and 0.82 for the CSF/serum ratio. Moreover, MR-proADM levels in CSF correlated with total protein concentrations, suggesting that increased CSF MR-proADM reflects, at least in part, disruption of the blood–CSF barrier and intrathecal inflammatory processes. These findings are not consistent with earlier work in bacterial meningitis and sepsis, where MR-proADM levels were associated with infection severity and mortality [22,23,24]. This suggests that in viral CNS infections, biomarker levels may not rise in the same manner as observed in bacterial infections. Importantly, the strong correlation between the first and second serum measurements (r = 0.83) indicates good analytical reproducibility and biological stability, supporting its potential application in longitudinal monitoring.

ADM is a multifunctional peptide with established vasoregulatory, anti-inflammatory, and antimicrobial effects [25]. Its stable fragment, MR-proADM, has been extensively validated as a biomarker of systemic infection, sepsis, and organ dysfunction [4,5,8]. ADM emerges as a critical mediator of endothelial integrity and vascular homeostasis, with potentially significant implications for neurological disease pathology. As a potent vasodilatory peptide, ADM signals through the calcitonin receptor-like receptor (CLR) coupled with receptor activity-modifying proteins (RAMPs), providing protection against endothelial dysfunction in multiple disease models [26]. In the context of systemic inflammation, ADM plays a key role through its ability to maintain vascular integrity while simultaneously modulating immune responses and reducing inflammation [27].

Given that blood–brain barrier dysfunction represents a critical initiating event in neuroinflammatory disease progression, including those associated with viral encephalitis, ADM signaling may offer therapeutic potential by stabilizing endothelial tight junctions and reducing microglial activation. The maintenance of endothelial barrier integrity through enhanced CLR/RAMP signaling could theoretically prevent the extravasation of peripheral immune cells and pro-inflammatory mediators into the brain parenchyma, thereby limiting the amplification of neuroinflammatory cascades [28]. Furthermore, by promoting vascular integrity and reducing inflammatory mediator production, ADM may attenuate the recruitment and polarization of microglia toward pro-inflammatory phenotypes, similar to protective mechanisms observed with other endothelial-targeted interventions [29]. Additionally, ADM analogs delivered via novel formulations may provide sustained therapeutic effects, highlighting the potential of ADM-based interventions in conditions with BBB breakdown and neuroinflammation [26]. Importantly, in our study we observed lower serum MR-proADM levels in TBE patients, which could indicate a reduced endogenous neuroprotective response, potentially contributing to subsequent neurological complications.

Previous studies in viral infections such as dengue and COVID-19 reported elevated MR-proADM levels, supporting the concept that ADM is actively involved in host defense and immune modulation within the CNS [8,30]. However, in our TBE cohort, we observed lower serum MR-proADM levels, while CSF MR-proADM levels correlated with total CSF protein (r = 0.53), a marker of blood–CSF barrier permeability. This finding suggests that, unlike in systemic viral infections or bacterial sepsis, TBE may be associated with an inadequate or blunted endogenous MR-proADM response, potentially reflecting a compromised neuroprotective mechanism during CNS infection.

MR-proADM has been only scarcely investigated in CSF in CNS infections. In the recently published multicenter retrospective study, the authors analyzed CSF ADM concentrations in acute meningitis and compared them with non-infectious neurological disorders [5]. However, the study grouped inflammatory etiologies together, including both viral and bacterial cases. This approach may obscure pathogen-specific differences, as our findings indicate that TBE is characterized by markedly low CSFADM concentrations, which could lead to misinterpretation if analyzed alongside other infections.

We propose several hypotheses to explain this phenomenon. One possibility is that TBEV has unique, previously undescribed effects on endothelial or neuroinflammatory pathways, resulting in reduced ADM production or release. Since no studies to date have compared ADM concentrations across different viral encephalitides, it is not yet possible to determine whether this pattern is specific to TBE. This question should be addressed in future research.

Another hypothesis that may explain our findings is the saturation of transport mechanisms responsible for carrying MR-proADM into the cerebrospinal fluid. Once serum concentrations exceed a certain threshold, further transfer into the CSF may no longer occur efficiently. This could account for the disproportionately low CSF levels observed in relation to the corresponding serum concentrations. This mechanism remains speculative, as specific ADM transport pathways into the CSF have not been described.

Emerging mechanistic considerations may also explain the unexpectedly low MR-proADM levels observed in our TBE cohort. TBEV is known to induce an exceptionally strong type I interferon (IFN-I) response, which represents a critical antiviral defense mechanism but can simultaneously exert suppressive effects on several endothelial and G-protein–coupled receptor pathways [31,32]. Notably, IFN-I signaling has been shown to downregulate components of the ADM receptor system, including selected RAMP isoforms and associated CLR regulatory networks, thereby attenuating ADM-mediated vasoprotective signaling [33,34]. Such IFN-I–driven repression of CLR/RAMP expression could result in diminished ADM biosynthesis or impaired receptor activation during TBEV infection, ultimately manifesting as the reduced circulating MR-proADM levels detected in our patients. This mechanism provides a biologically plausible explanation linking the characteristic antiviral response in TBEV to a blunted endogenous ADM pathway.

The development of new therapeutic agents for debilitating neurological conditions, including various forms of encephalitis (e.g., Japanese encephalitis [35], Nipah virus infection [36], autoimmune encephalitis [37]), is a major focus for global health and research funding. A critical challenge in this area is overcoming the blood–brain barrier (BBB) to effectively deliver therapeutic agents to the CNS [35]. Exploring ADM as a direct therapeutic target or as a modulator of therapeutic efficacy aligns perfectly with these priorities. Given AM’s established roles in vascular tone, cellular growth, and anti-inflammatory properties [4,38], investigating its potential as an exogenous therapeutic agent or through the modulation of its receptor signaling (AM1R, AM2R [39]) represents a promising avenue. Research into how AM signaling can restore metabolic balance, such as glutamine-glutamate metabolism, and reduce oxidative stress, as observed in Japanese encephalitis [40], also aligns with the development of therapies that target fundamental disease mechanisms. This research could lead to the development of novel neuroprotective drugs, advanced drug delivery strategies, and ultimately, improved treatment outcomes for patients suffering from the devastating effects of encephalitis.

The dual antimicrobial and anti-inflammatory properties of ADM make it plausible that it participates in local neuroimmune regulation during viral infection. Lowered MR-proADM may therefore reflect an lack of endogenous neuroprotective response aimed at limiting tissue injury caused by inflammation and oxidative stress.

Among the parameters examined, the CSF/serum MR-proADM ratio—representing the relationship between MR-proADM concentrations in CSF and serum—yielded the highest diagnostic performance (AUC = 0.816). A cut-off of >1.10 provided balanced sensitivity (79%) and specificity (80%). This ratio may better capture CNS-specific processes by normalizing for systemic levels, thus reflecting intrathecal production or selective passage through the blood–CSF barrier. The relatively high discriminative capacity observed here supports further validation of CSF/serum MR-proADM ratio as a biomarker in viral encephalitides.

TBEV infection triggers a complex immune response involving cytokine release, endothelial activation, and infiltration of immune cells into the CNS [18]. ADM synthesis is known to be upregulated in response to pro-inflammatory cytokines (e.g., TNF-α, IL-1β) and hypoxia, leading to vasodilation and reduced vascular permeability [41,42]. In our study, CSF MR-proADM levels correlated with total CSF protein, suggesting that local ADM production or trans-barrier leakage may act as a compensatory mechanism to preserve microvascular homeostasis and limit inflammatory damage. In contrast, serum MR-proADM levels were decreased in TBE patients, potentially indicating insufficient systemic neuroprotection. This imbalance may contribute to the development of long-term neurological or psychiatric sequelae, such as headache, vertigo, or cognitive disturbances.

TBEV exhibits pronounced neurotropism, which underlies the characteristic biphasic clinical course and the broad spectrum of neurological manifestations associated with the infection [43]. Following initial replication in cutaneous Langerhans cells and subsequent systemic dissemination, the virus gains access to the central nervous system, where it preferentially targets neuronal populations across multiple regions, including the anterior horn, brainstem, cerebellum, dentate nucleus, Purkinje cells, and striatum [12,43]. This selective affinity for neural tissue explains the wide range of clinical presentations—from mild meningitis to severe encephalitis accompanied by myelitis, flaccid paralysis, and cranial nerve dysfunction. The ability of TBEV, particularly the Siberian subtype, to persist within neural structures and induce chronic progressive encephalitis further underscores its potent neuroinvasive capabilities. Together, these features position TBEV as a highly neurotropic pathogen capable of causing both acute neuronal injury and long-term neurological sequelae [44].

The practical implications of our findings are twofold. First, serum MR-proADM could serve as a rapid, minimally invasive biomarker supporting the differentiation of infectious versus non-infectious neurological syndromes. Second, the CSF/serum ratio may represent a novel indicator of blood–CSF barrier dysfunction and neuroinflammatory activity. Combined, these markers could enhance early diagnosis and risk stratification in TBE. In the future, longitudinal assessment of MR-proADM could also help monitor recovery and predict long-term neurological sequelae, which remain a major burden in TBE survivors.

Several limitations should be acknowledged. The study was based on a relatively small cohort (*n* = 34) and retrospective design, which limits the generalizability of the findings. The lack of comprehensive cytokine profiling precludes mechanistic conclusions regarding ADM regulation. Furthermore, samples were collected at variable times during hospitalization, which may influence measured concentrations. Also, the control group consisted of patients with excluded neurologic and inflammatory diseases, although with some transient symptoms like headache, that could potentially influence on the results. Finally, multiple comparisons were performed, and exploratory results should be interpreted cautiously until confirmed in independent cohorts. Other limitation of the present study is the absence of serum and CSF samples from healthy donors, which would provide an ideal reference point. However, lumbar puncture is an invasive procedure and is not routinely performed in healthy individuals due to potential complications and lack of clinical indication. Consequently, our control group consisted of individuals who underwent lumbar puncture as part of routine differential diagnostics but in whom meningitis was ultimately excluded and who were free of comorbidities that could influence MR-proADM levels. It should be noted that this study is of a pilot nature, and future research will aim to expand both the patient and control cohorts to strengthen the findings.

## 5. Conclusions

In summary, MR-proADM concentrations are altered in both serum and CSF of patients with TBE and correlate with CSF protein levels, reflecting blood–CSF barrier involvement. Both serum MR-proADM and its CSF/serum ratio demonstrated promising diagnostic performance, supporting their potential utility as biomarkers of CNS infection and inflammation. Future studies should validate these findings in larger, prospective cohorts, evaluate longitudinal dynamics, and explore the mechanistic role of ADM in neuroimmune regulation during viral encephalitis. Despite the availability of effective vaccines, the rising incidence and expanding geographic range of TBE underscore the need for improved diagnostic tools.

## Figures and Tables

**Table 1:** Characteristics of MR-proADM levels in study groups.

Group	MR-proADM Level [pmol/L]	*n*	Mean	Median	Min	Max	SD
TBE	Cerebrospinal fluid 1 (CSF1)	20	730.74	666.62	101.00	1678.79	383.68
	Serum 1 (SER1)	20	402.00	357.65	130.42	1091.42	243.37
	Cerebrospinal fluid 2 (CSF2)	20	552.76	530.87	276.37	1149.48	223.03
	Serum 2 (SER2)	20	645.81	633.33	335.39	1201.70	225.56
Control	Cerebrospinal fluid 1 (CSF1)	14	709.13	780.49	102.00	1437.59	370.59
	Serum 1 (SER1)	11	2534.71	725.99	256.95	20,898.60	6099.333

## Data Availability

The data presented in this study are available on request from the corresponding author. The datasets generated and analyzed during the current study are not publicly available due to patient confidentiality and ethical restrictions. However, anonymized data supporting the findings of this study are available from the corresponding author upon reasonable request.
